# Wood-Based Cellulose Nanofibrils: Haemocompatibility and Impact on the Development and Behaviour of *Drosophila melanogaster*

**DOI:** 10.3390/biom9080363

**Published:** 2019-08-13

**Authors:** Pawan Kumar Mishra, Adam Ekielski, Sumit Mukherjee, Swetapadma Sahu, Saptarshi Chowdhury, Monalisa Mishra, Sushama Talegaonkar, Lubna Siddiqui, Harshita Mishra

**Affiliations:** 1 Department of Wood Processing Technology, Mendel University in Brno, 61300 Brno, Czech Republic; 2 Department of Production Management and Engineering, Warsaw University of Life Sciences, 02-787 Warsaw, Poland; 3 Neural Developmental Biology Lab, Department of Life Science, National Institute of Technology, Rourkela, Odisha 76908, India; 4 Biotechnology Department, Heritage Institute of Technology, Kolkata 700107, West Bengal, India; 5 Department of Pharmaceutics, School of Pharmaceutical Education and Research, Jamia Hamdard, New Delhi 110062, India; 6 Department of Pharmaceutics, Delhi Pharmaceutical Sciences and Research University, Govt. of NCT of Delhi 110017, New Delhi, India

**Keywords:** CNF toxicity, Drosophila melanogaster, haemocompatibility, wood-based CNF

## Abstract

Wood-based cellulose nanofibrils (CNF) offer an excellent scaffold for drug-delivery formulation development. However, toxicity and haemocompatibility of the drug carrier is always an important issue. In this study, toxicity-related issues of CNF were addressed. Different doses of CNF were orally administered to Drosophila and different tests like the developmental cycle, trypan blue exclusion assay, larva crawling assay, thermal sensitivity assay, cold sensitivity assay, larval light preference test, climbing behaviour, nitroblue tetrazolium (NBT) reduction assay, adult phenotype, and adult weight were conducted to observe the impact on its development and behaviour. A haemocompatibility assay was done on the blood taken from healthy Wistar rats. In Drosophila, the abnormalities in larval development and behaviour were observed in the behavioural assays. However, the cytotoxic effect could not be confirmed by the gut staining and level of reactive oxygen species. The larvae developed into an adult without any abnormality in the phenotype. The CNF did cause loss of weight in the adult flies and did not cause much toxicity within the body since there was no phenotypic defect. Hemolysis data also suggested that CNF was safe at lower doses, as the data was well within acceptable limits. All these results suggest that cellulose nanofibres have no significant cytotoxic effects on Drosophila. However, the developmental and behavioural abnormalities suggest that CNF may act as a behavioural teratogen.

## 1. Introduction

Plant-based polymers offer an environmentally benign alternative to inorganic materials for biomaterial applications [1,2,3,4,5]. Most of the cellulose in nature is produced by plants, where it serves as the main component of the cell wall. It is also found in some tunicates, bacteria and several algal species [6]. The term ‘nanocellulose’ is assigned to processed cellulose or cellulosic extracts that have a well-defined nano-scale structure [7,8]. The family of nanocellulose is divided mainly into three types: cellulose nanofibre, cellulose nanocrystals (CNC, also called cellulose whiskers), and bacterial cellulose (BC), also known as microbial cellulose [9,10]. The application of plant-based cellulose derivatives in biomedical applications is not new. They have been traditionally used as an excipient in tablets, in capsule shell preparation, as the formulation component of medicated patches, formulation coating, and many others [11,12,13,14]. However, most of these formulations were either for the oral route or for topical applications [15]. The nano-sized cellulose fibrils have created new opportunities for developing new formulations due to a large surface area and better properties. A wide range of formulations like tablets, aerogels, hydrogels, films, and patches have been developed using plant-based nanocellulose. However, Biocellulose (bacterial nanocellulose) has been the material of choice for biomedical applications of nanocellulose due to its “cleaner” nature [16,17]. This choice is additionally due to lack of data on toxicity, haemocompatibility, and genotoxicity studies.

Several studies have tried to address the toxicity concern of wood-based cellulose nanofibrils (CNFs) using cell-lines and other methods [18,19]. Oropharyngeal aspiration nanofibrillated cellulose (NFC) in mice resulted in reduced cell viability, triggered pro-inflammatory cytokines, and innate immune pathways in mice after oropharyngeal aspiration [20]. In another study, NFC caused genotoxic and inflammatory effects and DNA damage to the lung cells of mice [21]. CNF further caused peritoneal inflammation [19]. In *Daphnia magna* CNF displayed a low-toxic potential, indicating a low environmental risk factor [22]. The current study used the Drosophila model to check the toxicity of CNF.

In our previous studies on the preparation and application of plant-based nanocellulose aerogel in drug delivery, the candidature of CNFs as a scaffold for customized oral drug delivery was assessed [8,23]. In the current study, we have addressed the toxicity concerns of CNFs by an in vivo investigation using *Drosophila melanogaster* as a model organism and haemocompatibility using the blood from healthy Wistar rats. The 75% similarity with the disease gene, fully sequenced genome [24], and short life span has made Drosophila an ideal model to check the toxicity of CNF [25]. Additionally, Drosophila is used in various studies to check the toxic effect of nanoparticles [2]. Various behavioural, morphological, and biochemical parameters were checked in this study, after feeding the flies with cellulose nanofibres via the oral route.

## 2. Materials and Methods

### 2.1. Materials

Trypan blue, nitro blue tetrazolium, glacial acetic acid, glycerol, propionic acid, and methylparaben were obtained from Himedia. The chemicals were used as obtained without any further purification. The CNFs were procured from the U.S. Forest Service’s Cellulose Nanomaterials Pilot Plant at the Forest Products Laboratory (FPL, University of Maine, Orono, Maine, USA). The source of the cellulose for the preparation of these CNFs was specified as wood pulp. As per the specifications provided, the material supplied was 98 wt % dry powder, with a nominal fibre diameter of 50 nm and length of several microns. The surface of the fibres were hydrophilic, with Brunauer, Emmett, and Teller (BET) surface area being 31–33 m^2^/g.

### 2.2. Fly Management

The Drosophila stock (*Oregon R*) was obtained from the C-CAMP Fly Facility, Bangalore, India. For rearing the flies, standard food was prepared using yeast, sucrose, corn meal, and type I agar. Methyl paraben and propionic acid were added to inhibit microbial and fungal growth, respectively.

### 2.3. Treatment

The experimental setup contained one control vial and six different vials of flies treated with various concentrations of CNF (5, 10, 20, 50, 100, 200 mg/L). CNF obtained in the powder form was dispersed in Milli-Q water and sonicated using a probe sonicator two times and 15 min each. The dispersed NPs were added to the food in the desired concentrations. Once the food was dried, adult flies (five females and three males) were transferred to the vials. Flies were reared in 25 °C constant temperature, 70% humidity, and 12 h of light/dark conditions.

### 2.4. Developmental Cycle

The developmental cycle of both treated and control flies were checked and compared to see where there were any alterations. The development of the flies was checked every 6 h through various developmental stages. The percentage of adults hatchability from pupa was also counted to determine any developmental delay or damage [26].

### 2.5. Trypan Blue Exclusion Assay

Trypan blue assay is a simple test that differentiates the live and the dead cells. It is used to detect cell death in the larval gut. For this assay, the protocol of Krebs and Feder [27,28] was followed with slight modification. From each vial, five third instar larvae were taken and washed with 1X PBS to remove any food particles attached to the larval body. Then the larvae were transferred into a 0.02% solution of trypan blue and kept for 30 min in the dark. After the incubation, the excess stain was washed off with PBS (1X). The larvae were analyzed by using a stereomicroscope, and the images were taken to check any abnormality in the gut.

### 2.6. Larva Crawling Assay

The larval crawling behaviour is a simple assay that helps to understand the rhythmic behaviour of the larva and to detect any neural defects [29,30]. Larval behaviour was checked in the third instar larvae. For this, five third instar larvae were transferred onto a solid surface of a Petri plate made by using 2% agarose on which the larvae were allowed to crawl [30]. Graph paper was kept in the background to keep track of the path covered by each larva. The crawling of the larva was recorded with the help of a camera, and the total distance travelled was calculated by marking the grid lines of the graph paper. The average speed was measured in millimetres per second.

### 2.7. Thermal Sensitivity Assay

Temperature sensitivity in the flies is regulated by heat sensory neurons, and the temperature sensitivity assay is a simple assay performed to check any damage in those neurons. Thermal sensitivity assay was performed by following Mishra and Barik [31]. Briefly, 15 third instar larvae were taken and washed with 1X PBS. Next, they were transferred to a Petri plate containing 2% agar. The Petri plate was floated on hot water (40–45 °C) and kept in a beaker for at least 5 min to check the behaviour of the larvae. The larvae that climbed to the lid of the Petri plate were considered more sensitive to the temperature. The number of such larvae was counted.

### 2.8. Cold Sensitivity Assay

The cold sensitivity assay was carried out as per the protocol described by Mishra and Barik. [31] Briefly, 15 third instar larvae were taken in an agar plate, and the plate was floated on ice-cold water (14–18 °C) for 1 min to allow them to acclimatize to the environment. After 1 min, the larval movement was observed for 5 min. The control larvae showed sluggish movement and hesitated to move as they could sense the cold temperature. The movement of the treated larva was compared with the control, and the number of slow-moving larva were counted in each case.

### 2.9. Larval Light Preference Test

This experiment detected the early photoreceptor defect and was performed by following the method described by Sabat et al. [32]. For this, a Petri plate was divided into four quadrants, and the alternate quadrant was painted black. Than 1% agarose was poured on it and allowed to solidify. 12 third instar larvae were placed in the dark for 6 h before the experiment. Larvae were transferred to the agar plate, and the lid was closed having the same marking as the Petri plate. The Petri plate was kept under homogenous light, and the larvae were allowed to crawl for 5 min between the alternate transparent and black quadrants. After 5 min, the lid was opened, and the number of larvae in each quadrant was counted. The test was performed three times for each set of larvae.

### 2.10. Climbing Behaviour

The climbing assay was used to detect the locomotory defect by following Priyadarshini et al. [33]. Briefly, thirty adult flies were taken from the treated and control vials. Flies were transferred to a 100 mL measuring cylinder. A mark was made at 80 mL, which was measured at 16 cm. A cotton plug was placed at the opening of the measuring cylinder to avoid the escape of the flies. Flies were transferred to the measuring cylinder and allowed to settle for 10 min. Flies were brought to the bottom by gently tapping the cylinder three to four times. The climbing of the flies was recorded up to 10 s, and the number of flies that crossed the 80 mL mark was counted and noted. The experiment was repeated six times [30].

### 2.11. Nitroblue Tetrazolium Reduction Assay

To detect the amount of reactive oxygen species (ROS) in the larval hemolymph due to different concentrations of cellulose, the nitroblue tetrazolium reduction assay (NBT assay) was performed by following the method suggested by Sabat et al. [4] In this assay, samples having ROS were incubated with the yellow coloured dye, nitroblue tetrazolium (Y-NBT). After incubation, the dye was reduced to water-insoluble formazan particles (NBT) by the superoxide molecules that coloured blue. The absorbance was measured at 595 nm, which was proportional to the amount of ROS molecules. Briefly, 15 third instar larvae were taken, washed with PBS to remove the food particles and punctured at the thoracic region with a needle to extract hemolymph. The hemolymph extraction was performed on ice to avoid melanization. 5 μL of hemolymph was mixed with 10 μL of 1X PBS to make a total volume of 15 μL. After this equal volume of NBT solution was added to it. The mixture was left for 1 h in the dark and at room temperature. The reaction was stopped by adding 30 μL of 100% glacial acetic acid. It was centrifuged at maximum speed for 1 min. The absorbance was measured at 595 nm after the addition of 50% acetic acid [34].

### 2.12. Adult Phenotype

The adult flies were checked for phenotypic abnormalities. To analyze the phenotypic abnormalities i.e., whether there was any defect in the wing, eye, and bristle, 30 individual flies from the control as well as the treated vials were observed under a stereomicroscope.

### 2.13. Adult Weight

The adult flies were checked for any change in their body weight. For this, 50 flies (25 male and 25 female) were taken from each of the treated and control groups, anaesthetized and the weight was measured using a weighing balance (Act). The total weight of the flies from each group was checked and noted.

### 2.14. Haemocompatibility Assay

Haemocompatibility of CNF was determined at concentrations of 100, 250, 500, and 1000 µg/mL with a slight modification in the previously reported method [35]. Five mL of whole blood from healthy Wistar rats were collected in EDTA (ethylene diamine tetraacetic acid) tubes to avoid coagulation. Erythrocytes were collected by centrifugation at 500 g for 10 min at 4 °C and were thoroughly washed with physiological saline solution (PSS). Then erythrocyte stock solution (ESD) of 50% hematocrit was prepared in PSS. Haemocompatibility of the samples, including negative control—PSS, positive control—Double distilled water and previously mentioned CNF concentrations was determined using ESD. To 3 mL of each sample, 100 µL of ESD was added and incubated at 37 °C for 1 h, followed by centrifugation at 1000 g for 15 min at 4 °C. The absorbance of haemoglobin present in the supernatant was measured at 540 nm using a ultraviolet (UV)-Visible spectrophotometer (UV-1601, Shimadzu Corporation, Japan). Each sample was run in triplicates. The % hemolysis was determined using the following formula (Equation (1)):

% Hemolysis = ([A]_s_ − [A]_n_)/([A]_p_ − [A]_n_) × 100 (1)

where [A]_s_ = Absorbance of the sample under test, [A]_n_ = Absorbance of negative control, i.e., PSS, [A]_p_ = Absorbance of positive control, i.e., Double distilled water.

### 2.15. Statistical Analysis

All the experiments were performed in triplicates for all the concentrations, and the results were statistically analyzed using Graph pad prism 5.0 software. The statistical analysis was done with a two-tailed unpaired Student’s *t*-test with a 95% confidence interval. The results of the treated flies were compared with the control, and the data were represented in Mean ± SEM values. *p* < 0.5 was considered significant. For haemocompatibility studies, Statistica 11 software (TIBCO Software Inc., USA) was used, and significance was determined at a confidence interval of 95% (*p* < 0.5) by a one-way ANOVA (Analysis of Variance) followed by a Tukey HSD post hoc test.

## 3. Results

**Figure 1:**
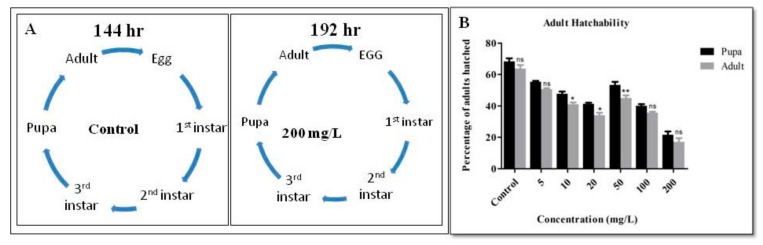
(**A**) The developmental cycle of control flies compared to the highest concentration of Wood-based Cellulose Nanofibrils (CNF) treatment, a delay of about 48 h was observed. (**B**) Percentage of the adult flies hatched from the pupa in different concentration of treatment vials compared to the control.

The toxicity of cellulose nanofibers can be expected to arise from procedures of synthesis/extraction (chemical, mechanical or enzymatic) and its unintended functionalization. The classical regenerated cellulose (microcrystalline cellulose and others) used in tableting and other dosage forms are mainly composed of cellulose II, in contrast to the naturally occurring cellulose I. On the other hand, CNFs are commonly produced by mechanical methods (not involving cellulose dissolution hence Cellulose I) and have been reported to have a minor amount of Lignin also, therefore source and process specific CNFs require independent toxicity testing [8]. *Drosophila* proved to be a model organism to detect the toxicity nanoparticles taken orally [36]. The developmental cycle of the flies was observed to analyze the developmental defect. It was found that the duration of the 3rd instar larvae took three days in the control and treated, whereas the development of pupae took 48 h in the control and 72 h in the treated flies. The development of the adults from the pupae was about 48 h in the control, 5 and 10 mg/L concentration, whereas in other concentrations the time taken was 72 h. Flies treated with CNF showed a delay in the developmental cycle at the highest concentration. This developmental delay may be associated with ovarian defects, delay in the egg chamber development or disturbance in the oogenesis period, which was already reported in other NPS [34,36]. Adult hatchability percentage was counted from all the vials. It was observed that the number of pupae was less as the treatment concentration increased, whereas there was a slight decrease in the hatchability percentage (Figure 1).

**Figure 2:**
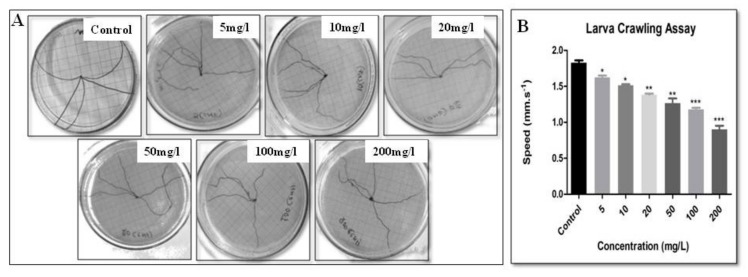
Larval crawling assay to check the speed of the larvae and trailing path. (**A**) Trailing path of the larvae. (**B**) Distance traveled by larva in millimeters per second (* for *p*-value < 0.05, ** for *p*-value < 0.001, *** for *p*-value < 0.0001).

Larvae move by contracting their body, which creates a wave to move from one place to another. These body contractions are controlled directly by the motor neurons that are present in the brain of the larva. Therefore, if there is any abnormality in the neurons, it is reflected as a defective crawling pattern of the larvae. As observed in the control larvae, the crawling speed was 1.52 ± 0.087 mm s^−1,^ which increased with an increase in concentration and reached 0.90 ± 0.053 mm s^−1^ at the highest concentration. The larvae trailing paths are demonstrated in Figure 2. The larval crawling assay is a ruler to check the functionality of neurons [37,38]. NPs altering the function of neurons was recently reviewed by Barik and Mishra [34,39,40]. CNF-treated larvae also show decreased larva crawling speed; with a mild alteration in crawling path suggesting that CNF alters the neurons of the larvae.

**Figure 3:**
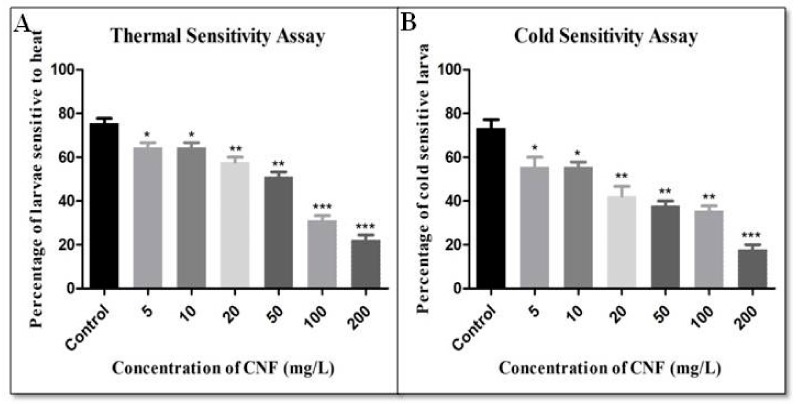
Temperature sensitivity assay to check the thermal sensing ability of the larva. (**A**) Heat sensitivity of larva treated with various concentrations of Wood-based Cellulose Nanofibrils (CNF). (**B**) Cold sensitivity of larva treated with various concentrations of CNF (* for *p*-value < 0.05, ** for *p*-value < 0.001, *** for *p*-value < 0.0001).

The larvae that were more sensitive to the temperature tended to move fast and climb up to the lid of the Petri plate to avoid the heat. The larvae that had defective thermal sensitivity did not show any avoidance. They stayed on the Petri plate and eventually died if kept for a longer time in the heat. In the control larvae, 75.55 ± 2.223% of larvae were sensitive to heat, whereas in 5, 10, 20, 50, 100 and 200 mg/L concentrations, the percentage was found to be 68.89 ± 2.220%, 64.45 ± 2.223%, 57.78 ± 2.223%, 51.11 ± 2.220%, 31.11 ± 2.220% and 22.22 ± 2.223%, respectively (Figure 3). *Drosophila* larvaa also able to sense the cold. Cold sensitivity was demonstrated by the larvae based on their sluggish behaviour and hesitation to move. In this assay, it was found (Figure 3) that in the control, 73.33 ± 3.848% of larvae were sensitive to cold as observed by their slow movement. In 5 and 10 mg/L, 55.56 ± 4.443% and 55.55 ± 2.223% larvae were cold sensitive. In 20 and 50 mg/L, the cold sensitivity was found to be 42.22 ± 4.447% and 37.78 ± 2.223% of larvae, whereas in 100 and 200 mg/L the percentage was 35.55 ± 2.223% and 17.78 ± 2.223%, respectively (Figure 3). [41]. To further study the effect of CNF, the temperature sensitivity assays were performed in the third instar larvae. Temperature sensitivity in *Drosophila* is mediated by transient receptor potential (TRP) channels (painless and TRPA1) [42,43,44]. Thus, any alteration in the TRP channel results in the fluctuation of temperature [45,46]. The range of temperature varied from 18 to 28 °C. Beyond which the larva showed faster movement at a higher temperature and slow movement at low temperature. A decreased larval sensitivity towards temperature sensing was observed with the increasing concentration of CNF. These results were in agreement with an earlier silica nanoparticle study where the TRPM2 and TRPM8 channel of human cell lines were affected [46,47].

**Figure 4:**
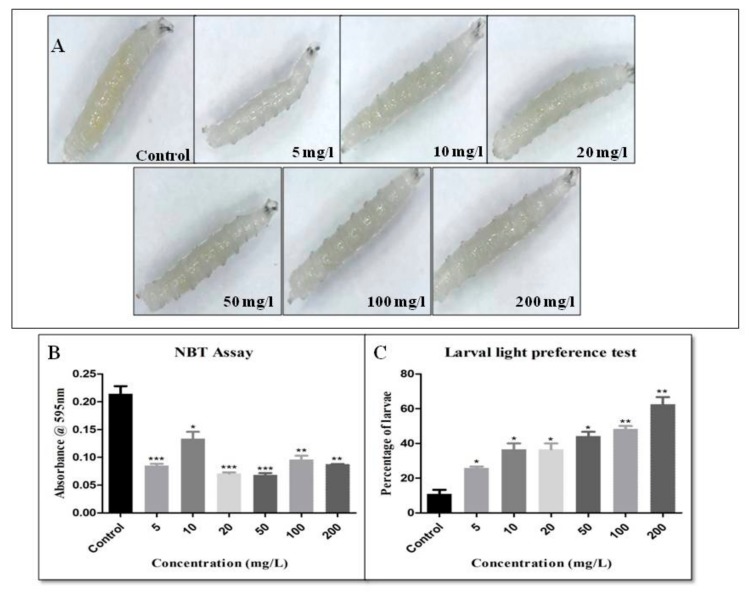
(**A**) Trypan blue assay of the larva to check cell damage in the gut. (**B**) NBT assay to determine the level of ROS in the larva at various concentrations of Wood-based Cellulose Nanofibrils (CNF) (**C**) Larval light avoidance behaviour to assess the percentage of larva in the light and dark region (* for *p*-value < 0.05, ** for *p*-value < 0.001, *** for *p*-value < 0.0001).

The light preference test of the larva was performed to observe any early defect in the light-sensing neurons. In this test, it was observed that the percentage of larvae that were attracted to the light increased with an increasing concentration of cellulose nanofibres. In the control, the percentage of larvae that were attracted to the light was 22.22 ± 2.777%. The percentage of light-sensitive larvae was 22.22 ± 2.777% in 5 mg/L, 19.45 ± 2.777% in 10 mg/L, 22.22 ± 2.777% in 20 mg/L, 27.78 ± 2.777% in 50 mg/L, 36.11 ± 2.780% in 100 mg/L and 44.45 ± 2.777% in 200 mg/L (Figure 4). Light avoidance behaviour of the larva is mediated by prothoracicotropic hormone (PTTH), which acts on Bolwig’s organ and class IV dendritic arborization [48,49,50]. With increasing CNF concentrations light preference behaviour of the larvae increased suggesting CNF interfere with light avoidance behaviour. Only the dead cells were stained by trypan blue. Thus, dead cells can be distinguished from the live ones. The larvae were tested to see any damage in the gut due to CNF uptake. It was found that all the larvae were normal and showed no damage in the gut after the intake of CNF (Figure 4). Trypan blue staining suggests that CNF does not cause major damage to the gut like other NPS [32]. This experiment suggested that CNF was non-toxic in comparison to the NPs. NPs like gold, silver, and titania are known to generate a significant amount of ROS within the body. The NBT assay detects the ROS produced in the hemolymph of the larvae. In the case of the control, the absorbance at 595 nm was 0.21 ± 0.014, which decreased to 0.09 ± 0.0003 at the highest concentration of 200 mg/L. As ROS production is directly proportional to the absorbance, thus it can be concluded that the amount of ROS generated, decreased with increasing concentrations of cellulose nanofibres (Figure 4). In the present study, the NBT assay showed that after CNF treatment, the level of ROS production reduced. Earlier studies reported increased ROS production in a higher concentration of cotton wool cellulose. At a lower concentration, nanocellulose caused a lower level of ROS production [41].

**Figure 5:**
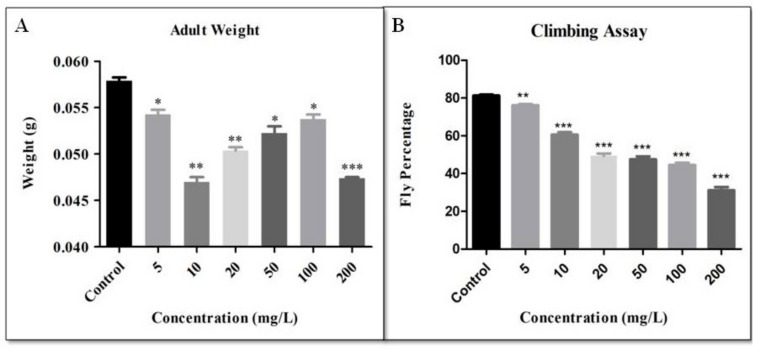
(**A**) Weight of 50 adult flies (25 males and 25 females) was measured and plotted on the graph (weight in grams). (**B**) Climbing assay to check the flies ability to climb up the walls of a measuring cylinder up to a certain distance (80 mL or 16 cm of a 100 mL measuring cylinder) (* for *p*-value < 0.05, ** for *p*-value < 0.001, *** for *p*-value < 0.0001).

**Figure 6:**
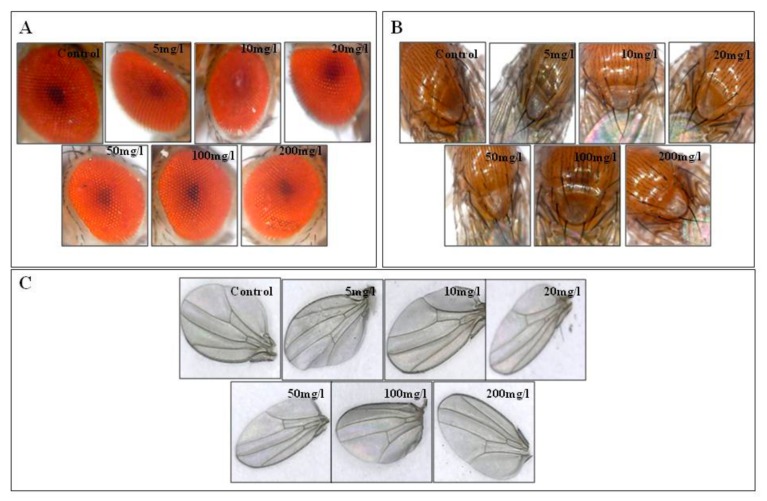
Adult phenotypes to check abnormalities in the (**A**) eye, (**B**) bristles and (**C**) wing. There were no such phenotypic defects observed after the treatment.

The climbing ability of the flies decreased with increasing concentration of CNF. Climbing assay demonstrates the change in the behaviour of the fly concerning gravity. The results were analyzed by the number of flies above the 80 mL mark in 10 s. The percentage of flies that climbed above the 80 mL mark in the control was 81.26 ± 0.638. In 5, 10, 20, 50, 100 and 200 mg/L, the fly percentage that climbed above the 80 mL mark was 76.20 ± 0.611, 60.60 ± 1.301, 49.38 ± 1.220, 47.58 ± 1.543, 44.58 ± 1.230 and 31.11 ± 1.800, respectively. The climbing behaviour was performed to check the functionality of the neurons of the adults. Flies prefer to climb against gravity. In gold, silver, titania, hydroxyapatite, and zirconia treated flies, positive geotropism was observed, which hinted at defective antennae [31,32,36,51,52]. The fly weight was measured from each vial. The weight of 50 (25 males and 25 females) control flies was found to be 0.06 ± 0.002 gm, whereas in 5 mg/L the weight was 0.05 ± 0.001 gm. Likewise the weight was 0.05 ± 0.002 gm in 10 mg/L, 0.05 ± 0.002 gm in 20 mg/L, 0.05 ± 0.001 gm in 50 mg/L, 0.05 ± 0.004 gm in 100 mg/L and 0.05 ± 0.0001 gm in 200 mg/L (Figure 5). The body weight of all the flies was plotted on a graph. Cellulose is often used in weight-reducing supplements to reduce the weight of obese individuals by clearing the gut [53,54]. In this study, it was found that fly weight decreased with various concentrations of CNF as compared to the control. Adult flies were checked under a microscopic camera to observe any phenotypic defect in the eye, wing, and bristle. No such defect was observed in the eye, wing, or the bristles (Figure 6).

The compatibility of drug carriers with blood components is a pre-requisite for establishing its application in therapeutics. Haemocompatibility assay indicates the level of hemolysis caused by a sample when exposed to red blood cells. A low level of % hemolysis suggests better compatibility of the drug carrier. The % hemolysis caused by 100 µg/mL of CNF was found to be 0.98 ± 0.120% and significantly changed to 4.07 ± 0.220% at 1000 µg/mL of CNF. The levels were within acceptable limits and suggested that even at a very high concentration of CNF, % hemolysis was relatively low. Thus, they can be used as drug carriers.

**Figure 7:**
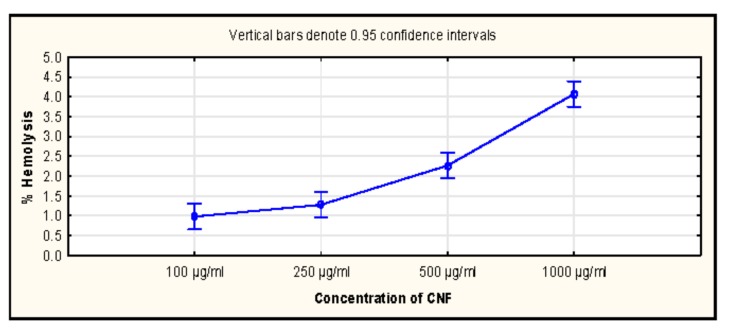
Hemolysis caused by different doses of wood-based cellulose nanofibrils (CNF) suspension.

**Figure 8:**
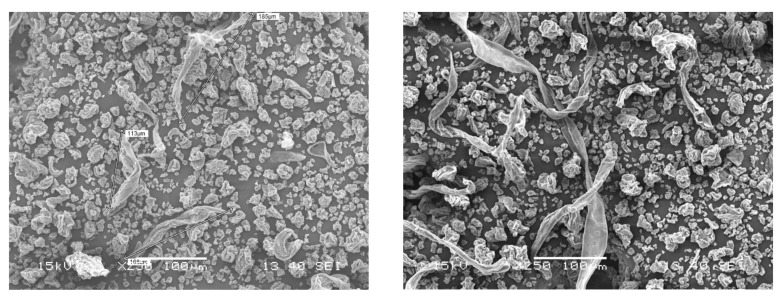
Representative Scanning Electron Microscopic (SEM) images of CNF used in this study.

Haemocompatibility data and representative SEM (Scanning Electron Microscopy) of CNFs used in this study can be found in Figure 7 and Figure 8, respectively. Heamocompatibility data also supports significant toxicity at higher doses. However, the hemolysis at lower doses was found be less than 2%. The increase in hemolysis from 100 to 250 μg/mL was not significant (*p*-value- 0.05). This data also supported the possibility of CNF utilization as a drug at lower doses only.

## 4. Conclusions

The current study investigated the effect of cellulose nanofibres on the development and behaviour of *Drosophila*. CNF was added directly to the fly food which was taken orally by the larvae once they hatched from the eggs. These nanoparticles enter into the gut and cause abnormalities in the larval development and behaviour as was seen in the behavioural assays. However, no cytotoxic effect was seen as indicated by the gut staining and level of reactive oxygen species. The larvae developed into adults where it showed no abnormality in the phenotype. The CNF reduced the weight of the adult flies. Hemolysis data also suggested CNF safety at lower doses. All these results have suggested that cellulose nanofibres have no significant cytotoxic or genotoxic effect on Drosophila. However, the changes in development and behaviour were observed at higher concentrations, which suggests that CNF may act as a behavioural teratogen and thus requires further investigation in other mammalian model organisms such as mice or rat. Therefore, from the aforementioned report, it can be inferred that a lower dose of CNF might be utilized as a potential drug carrier in various applications of the therapeutic industry.
